# Neutrophil-to-lymphocyte and platelet-to-lymphocyte ratio in Chinese Han population from Chaoshan region in South China

**DOI:** 10.1186/s12872-019-1110-7

**Published:** 2019-05-27

**Authors:** Lishan Wu, Shan Zou, Cantian Wang, Xuerui Tan, Min Yu

**Affiliations:** Department of Cardiology, the First Affiliated Hospital, Shantou University Medical College, Shantou, 515041 Guangdong China

**Keywords:** Neutrophil-to-lymphocyte ratio, Platelet-to-lymphocyte ratio, Reference range

## Abstract

**Background:**

Neutrophil-to-lymphocyte ratio (NLR) and platelet-to-lymphocyte ratio (PLR) are assumed to be prognostic factors in many diseases such as inflammatory diseases, cardiovascular diseases and cancer. However, NLR and PLR are race specific, it is important to determine the reference values of NLR and PLR in different races. The study aimed to investigate the reference range of NLR and PLR in Chinese Han population from Chaoshan region in South China.

**Methods:**

A retrospective study was conducted in the First Affiliated Hospital of Shantou University Medical College in South China. Five thousand healthy adults aged 20–69 years were included. NLR and PLR were determined.

**Results:**

Of 5000 healthy adults, 2500 men and 2500 women were included. The mean NLR and PLR across all ages for men and women were 1.59 ± 0.59, 92.88 ± 28.70, 1.62 ± 0.64 and 108.02 ± 32.99, respectively. The 95% reference range of NLR in normal male and female are 0.43~2.75 and 0.37~2.87, PLR are 36.63~149.13 and 43.36~172.68, respectively. The female had a higher NLR at age 30~49 than the male while the NLR at age 60~69 was higher in male than in female. The PLR was higher in female than in male.

**Conclusion:**

The study provides reference data on NLR and PLR from different age and sex groups in South China. NLR and PLR varied with age and sex.

## Background

Complete blood count (CBC) test is a simple economic and extensively used basic hematological test which mainly included white blood cell (WBC) count, red blood cell (RBC) count and platelet count. The most abundant white blood cells in healthy humans are neutrophils, which play important roles during acute and chronic inflammation and may be potential therapeutic targets in cardiovascular diseases [1]. Neutrophil-to-lymphocyte ratio (NLR) and platelet-to-lymphocyte ratio (PLR) are the proportions of absolute neutrophil to lymphocyte and platelet to lymphocyte counts retrieved from the CBC test. As markers of inflammation, various studies have demonstrated the correlation between NLR, PLR and many diseases such as inflammatory diseases [2], cardiovascular diseases [3], cancer [4] and long-term type 2 diabetes remission after metabolic surgery [5]. NLR and PLR are assumed to be prognostic factors. Although there have been extensive investigations on NLR and PLR, the normal ranges of NLR and PLR were less investigated. It was reported that the average NLR is 2.15 in the US population [6] and 1.65 in South Korea [7], which suggested that NLR is race specific. Therefore it is important to investigate the ranges of NLR and PLR for evaluating the prognostic role of NLR and PLR in different races. The aim of this study is to explore the reference values of NLR and PLR among the Han populations in Chaoshan District of Guangdong Province in South China.

## Methods

The study was conducted retrospectively in the First Affiliated Hospital of Shantou University Medical College in South China. CBC tests between February 2018 and July 2018 were reviewed and collected consecutively from healthy persons aged 20–69 years without diagnosed diseases including acute or chronic infection, heart failure, renal failure, autoimmune or hematopoietic diseases. The healthy adults were divided into groups according to gender and age. A total of 5000 healthy adults were included. Neutrophil, lymphocyte and platelet counts were determined by the Coulter method. NLR and PLR were calculated as the ratio of neutrophil cell and platelet count to lymphocyte cell count, respectively. The samples were excluded with WBC less than 3.5 × 10^9^/L or more than 9.5 × 10^9^/L and platelet less than 125 × 10^9^/L or more than 350 × 10^9^/L. The study was approved by the ethics committee of Shantou University Medical College.

Data are presented as mean ± SD. Differences between group means were assessed by an unpaired Student’s *t*-test for single comparisons or by ANOVA for multiple comparisons using SPSS 16.0. *P* value < 0.05 was considered significant.

## Results

**Fig. 1:**
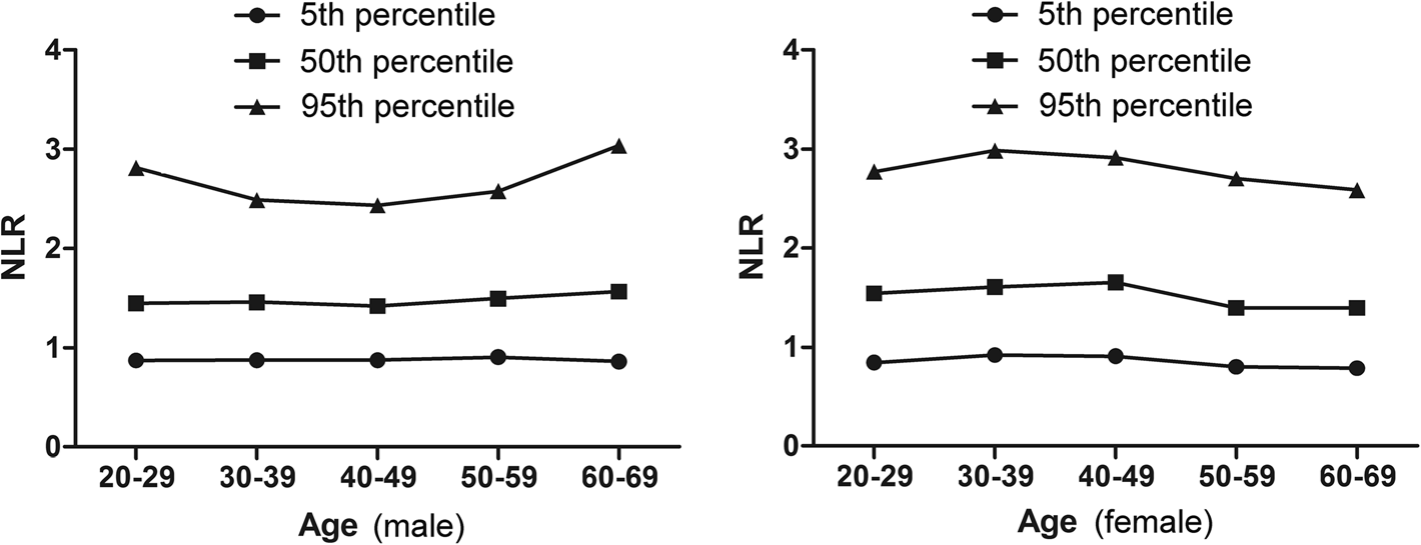
The percentile nomogram for NLR in male and female

**Fig. 2:**
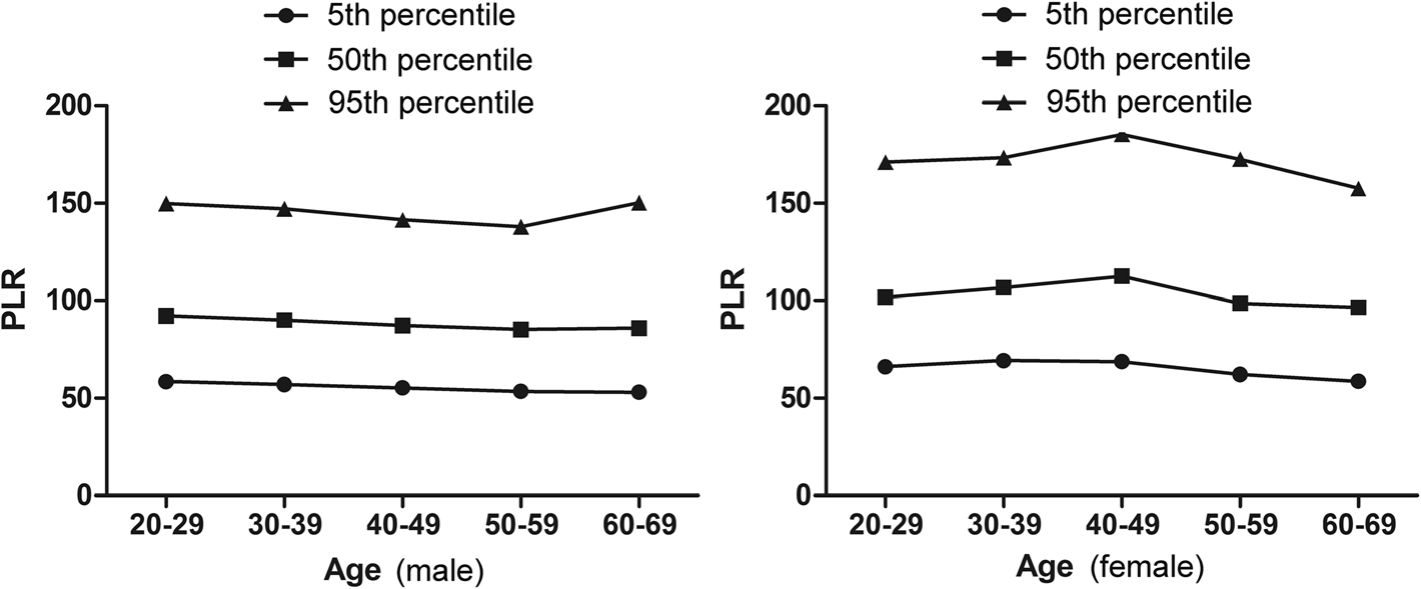
The percentile nomogram for PLR in male and female

In present study, there are 5000 CBC tests. Two thousand five hundred men and 2500 women were included. As shown in the Table 1, the differences of age, WBC counts, neutrophils (NE) counts and RBC counts between the male and female were not significant. The male had a higher lymphocyte (LY) counts and hemoglobin (HGB) than the female while the female had a higher platelet (PLT) counts, NLR and PLR. The mean NLR and PLR across all ages for men and women were 1.59 ± 0.59, 92.88 ± 28.70, 1.62 ± 0.64 and 108.02 ± 32.99, respectively. The 95% reference range of NLR in normal male and female are 0.43~2.75 and 0.37~2.87, PLR are 36.63~149.13 and 43.36~172.68, respectively. NLR and PLR were analyzed based on sex and age (500 cases in each group), which were showed in Figs. 1 and 2 and Tables 2 and 3. The female had a higher NLR at age 30~49 than the male while the NLR at age 60~69 was higher in male than in female. The NLR was affected by age. In female the NLR increased with aging at age 20–49 while decreased in age groups of > 50 years. There is a sex difference in PLR at age 30~59, with higher in female than in male. The PLR decreased in women older than 50 years.

**Table 1:** Main characteristics of the overall cohort based on sex

	Male	Female	*P* Value
Number	2500	2500	
Age (years, mean ± SD)	44.42 ± 14.02	44.45 ± 14.02	0.924
Mean WBC (×10^9^/L)	6.55 ± 1.28	6.04 ± 1.25	0.059
Mean NE (× 10^9^/L)	3.54 ± 0.93	3.34 ± 0.96	0.328
Mean LY (× 10^9^/L)	2.35 ± 0.60	2.18 ± 0.56	0.001
Mean NE/LY	1.59 ± 0.59	1.62 ± 0.64	0.002
95% reference range	0.43~2.75	0.37~2.87	
Mean RBC (×10^9^/L)	4.96 ± 0.41	4.50 ± 0.40	0.186
Mean HGB g/L	152.88 ± 11.59	134.45 ± 12.76	0.013
Mean PLT (×10^9^/L)	206.30 ± 40.81	222.22 ± 44.47	0.000
Mean PLT/LY	92.88 ± 28.70	108.02 ± 32.99	0.000
95% reference range	36.63~149.13	43.36~172.68

**Table 2:** Neutrophil-to-lymphocyte ratio at different groups

Subgroup (age)	Neutrophil-to-lymphocyteratio (male)	Neutrophil-to-lymphocyteratio (female)	*P* value
20~29	1.57 ± 0.61	1.64 ± 0.59	0.675
30~39	1.55 ± 0.56	1.72 ± 0.68^#^	0.004
40~49	1.53 ± 0.52	1.74 ± 0.61^#^	0.000
50~59	1.60 ± 0.55	1.52 ± 0.62^##^	0.223
60~69	1.71 ± 0.68^△^	1.51 ± 0.64^##^	0.005

^△^compared with group age 20~59 (*P* < 0.01) ^#^compared with group age 20~29 (*P* < 0.05)

^##^compared with group age 20~49 (*P* < 0.01)

**Table 3:** Platelet-to-lymphocyte ratio at different groups

Subgroup (age)	Platelet-to-lymphocyte ratio (male)	Platelet-to-lymphocyte ratio (female)	*P* value
20~29	96.16 ± 28.48	107.46 ± 31.15	0.176
30~39	94.91 ± 30.49	111.91 ± 32.93^#^	0.017
40~49	91.84 ± 26.84	116.41 ± 34.49^##^ *	0.000
50~59	89.82 ± 26.72^△^	103.80 ± 32.83^△△^	0.001
60~69	91.67 ± 30.38	100.53 ± 31.11^△△^	0.922

^△^compared with group age 20~39 (*P* < 0.01) ^#^compared with group age 20~29(*P* < 0.05)

^△△^compared with group age 30~49 (*P* < 0.01) ^##^compared with group age 30~39(*P* < 0.05)

^*^compared with group age 20~29 (*P* < 0.05)

## Discussion

In present study we measured the NLR and PLR in 5000 Chinese healthy adults. We found that the 95% reference range of NLR in normal male and female are 0.43~2.75 and 0.37~2.87, PLR are 36.63~149.13 and 43.36~172.68, respectively. The NLR and PLR vary by sex and age.

CBC, an economic and extensively used basic hematological test, included a hemogram and differential WBC count. Though the CBC test was usually used to the diagnosis of anemia, certain cancers, infection and immunodeficiencies, it has been recently found that some parameter of CBC such as NLR and PLR are associated with activity, morbidity and mortality in different diseases. In active rheumatoid arthritis [8], systemic lupus erythematosus [9] and Takayasu’s arteritis [10], NLR and PLR were significantly increased than that in the control and can be used to evaluate disease activity. In patients with hepatocellular carcinoma after hepatectomy, postoperative NLR and PLR were associated with recurrence [11]. Additionally, admission NLR can be used to predict worse outcomes and hospital mortality in patients with acute type A aortic dissection [12–15].

Though NLR was used widely in many diseases, the cut off points for risk stratification were arbitrary used in these studies, which did not consider the factors affecting the NLR such as the disease category, age, and race of patients. In the studies from western countries a higher cutoff value was suggested than that in Asian or African. In fact the NLR in the United States population was generally higher than Asian races. It was reported that NLR is 2.24 in Whites and 1.76 in Blacks in the United States [6] while 1.65 in South Korea [7] and 1.72 in central China [16]. The effects of sex on NLR varied with race. There was no significant difference with NLR between in men and women in the United States population [6] while significant in Asian [7, 16]. In present study, we found that the mean NLR across all ages was higher in female than in male, which is consistent with studies in other Asian countries [7]. The mechanisms for sex-related differences in NLR are not well known. Sex hormones such as estrogen level may be attributed to the difference. The female had higher estrogen level than the male. It had been found that estrogen can delay neutrophil apoptosis [17], which leaded to higher NLR in female. Though NLR was different between sexes, a study from central China showed that it is higher in male than in female [16] while it is the reverse in present study, suggesting there is regional variations in NLR.

NLR can be also affected by age, especial in female. Estrogen decreased dramatically after menopause [18]. Thus, it was not surprising that the NLR in women is higher in age groups of < 50 years than > 50 years [7, 19], which was also verified in present study.

Unlike NLR, the PLR is less investigated. There is also a sex difference in PLR, with higher in women than in men [7]. The difference may be associated with the higher platelet counts in women. Many studies have found that females have higher platelet count than males [20–23]. The mechanisms of sex-related difference in platelet count are also not well known. One explanation is that there is lower serum iron in menstruating and elder women, which stimulates platelet production [24–26]. Additionally, sex hormonal difference such as estrogen level may be also play a role. It was reported that estrogens favour platelets formation in mouse [27]. Apart from sex, platelet count varies by age, being higher in youth than in old age [28, 29], which may be associated with hematopoietic stem cell. In elderly people a reduction in hematopoietic stem cell reserve would lead to reduction of the platelets formation.

Though the female has a higher PLR than the male, another study from central China showed no difference between gender groups in PLR [16], suggesting regional variation of PLR in China.

A few limitations were apparent in present study. First, the study is a retrospective study and routine blood analyses were collected from healthy populations in the checkup center of hospital, the effects of chronic concealed inflamation and smoking [30] on NLR and PLR can not be excluded. Secondly, owing to the geographic difference of platelet counts [21, 31], the reference range of PLR in healthy population in Chaoshan region may be different from other regions in China.

## Conclusion

In summary, we found that the reference range of NLR and PLR in male was different from in female from Chaoshan region in South China. The NLR and PLR varied with age and sex.

## Data Availability

Raw data supporting the obtained results are available at the corresponding author.

## Abbreviations

CBC: Complete blood count
NLR: Neutrophil-to-lymphocyte ratio
PLR: Platelet-to-lymphocyte ratio
WBC: White blood cell
